# Twelve-Week Game-Based School Intervention Improves Physical Fitness in 12–14-Year-Old Girls

**DOI:** 10.3389/fpubh.2022.831424

**Published:** 2022-02-09

**Authors:** Tanja Petrušič, Nebojša Trajković, Špela Bogataj

**Affiliations:** ^1^Faculty of Education, University of Ljubljana, Ljubljana, Slovenia; ^2^Faculty of Sport and Physical Education, University of Niš, Niš, Serbia; ^3^University Medical Centre, Department of Nephrology, Ljubljana, Slovenia

**Keywords:** sport, adolescents, physical fitness, team games, school intervention

## Abstract

The aim of this study was to determine the effects of a twelve-week game-based school intervention on physical fitness in girls aged 12–14 years. Fifty-nine adolescent girls (13.2 ± 0.3 years) were randomly assigned to a group that participated in a game-based after-school program (EXP) or a control group (CON) that participated only in mandatory physical education. The EXP group had the additional program twice a week after school for 40 min/session for 12 weeks alongside with regular physical education classes. The EXP program consisted mainly of small-sided games of football, basketball, handball, and volleyball. The assessment included a physical fitness assessment with standardized tests for this age group: countermovement jump (CMJ), standing long jump, bent arm hang, overhead medicine ball throw, sit-ups for 30 s, and Yo-Yo Intermittent Recovery Level 1 Test (YYIRT1). There was a significant interaction between group (EXP vs. CON) and time (pre-test vs. post-test) for the standing long jump (*p* < 0.001), overhead medicine ball throw (*p* < 0.001), 30 s sit-ups (*p* = 0.030), bent- arm hang (*p* < 0.001), and YYIRT1 score (*p* = 0.004). In addition, a significant main effect was found for time in countermovement jump (*p* < 0.001). The results of this study indicate that the after-school game-based intervention significantly improves adolescent girls' physical fitness compared to regular physical education. The overall conclusion suggests that as few as two additional sessions per week are sufficient to produce significant changes in physical fitness in adolescent girls.

## Introduction

Physical fitness is considered as one of the most important predictor for healthy and active lifestyle (1–4). However, the level of physical fitness of children and adolescents has decreased significantly over the years (5, 6). Consistent participation in physical activities has been shown to be very important in young children and has great fitness benefits in both the short and long term (7). Some of these are reflected in body composition as well as physical, psychological, and social parameters (8). It is recommended that children engage in 60 or more minutes of moderate physical activity daily (9). More recent recommendations in children and adolescents include vigorous activities and thus recommending an average of 60 min/day of moderate-to-vigorous intensity aerobic physical activity across the week for providing health benefits (10).

Children spend most of their time at schools, mostly indoors (11), which could have impact on their physical fitness and accordingly overall health. Therefore, school-based interventions are probably the most effective way to improve physical fitness and to promote physical activity in children. Moreover, recent study showed that physical education and organized sport have significant contribution for the achievement of the physical activity recommendations (12).

Modified games can be a methodical approach that helps develop quite relevant tactical/technical knowledge in a short period of time (13). These games are better known as small-sided games (SSG). These games are thought to have their origins in the variety of games children used to play in the street, often being forced to change the rules to fit the game into the given space or to make it appropriate for the number of players available (14). There are few studies that have looked at the effects of SSG in children, however, their sample usually consisted of athletes (15, 16). Some studies that have focused on the effects of SSG in overweight children, as well as those that have used a specific program based on SSG for a specific sport, in most cases soccer (17, 18). Larsen et al. (19) investigated the effects of SSG in school on cardiovascular adaptations in children aged 8–10 years. The results showed that 10 months of SSG with 3 × 40 min per week during school lessons can have positive effects on cardiovascular health in children. In addition, an 11-month study showed that an SSG-based program increased VO2max by 9% and reduced body fat by 7% in children aged 15 years. Therefore, game-based training is considered to be a good strategy to contribute to the maintenance of a fitness level and a healthy lifestyle in children.

Increased participation in game-based training performed in the school setting may increase physical fitness performance in children and adolescents. However, there is only one study with school-aged children that showed that frequent low-volume ball games had positive fitness effects in 8–10-year-old children. Moreover, to the author's knowledge, there is not a single study that has examined the effects of game-based training on physical fitness in girls aged 12–14 years. Having in mind that girls tend to engage in less physical activity compared to their male peers, the aim of this study was to determine the effects of a 12-week game-based school intervention on physical fitness in girls aged 12–14 years. It was hypothesized that a game-based school intervention would improve physical fitness parameters in girls aged 12–14 years.

## Materials and Methods

### Subjects

The present study was a randomized experimental trial comparing a game-based after-school program with traditional physical education. Sixty-four adolescent girls (aged 12–14 years) were invited from a single school to participate in this study. Fifty-nine girls (13.2 ± 0.3 years) chose to undergo evaluation for the baseline testing. After the baseline testing, the girls were randomly assigned to a group that participated in a game-based after-school program (EXP) or a control group (CON) that participated only in compulsory physical education. The general characteristics of the participants are shown in Table 1. Before the intervention began, all participants and their parents or guardians were familiarized with the experimental procedures and signed an informed consent form. The study procedures were approved by the local ethics committee (Ref. No. 11/2019) and were conducted in accordance with the Declaration of Helsinki.

**Table 1:** General characteristics of the participants.

**Variable**	**EXP group**	**CON group**
	**(*n* = 30)**	**(*n* = 29)**
BH (cm)	163.9 ± 10.1	165.4 ± 9.4
BW (kg)	52.3 ± 9.9	54.1 ± 9.6
BMI (kg/m^2^)	19.2 ± 2.3	19.3 ± 1.9
Age (years)	13.3 ± 0.3	13.2 ± 0.4

*BH, body height; BW, body weight; BMI, body mass index; EXP, experimental; CON, control; n, number of participants. Values are defined as mean ± standard deviation*.

### Procedures

All tests were performed by the same investigators during a single visit after an overnight fast. The tests included an examination of body composition and physical fitness. Body height was measured to the nearest 0.5 cm using a wall-mounted stadiometer. Weight was measured on a calibrated beam scale with an accuracy of 0.1 kg.

Both groups participated in regular physical education classes twice a week for 45 min, with the EXP group additionally participating in the game-based training program. The game-based training sessions were led by students from the Faculty of Sport and took place twice a week after school for 40 min/session for 12 weeks. Each session began with a 5–7 min warm-up with moderate-intensity running and exercises relevant to the game/sport of that session. The main part of the session consisted of two different games played for 30 min. The main activity was followed by a 3–5 min cool-down exercises. The programme consisted mainly of small-sided football, basketball, handball, and volleyball games, which have been shown to have high participation and training intensity for all children (20). For practical reasons, different small games were occasionally played with different numbers of players and on different sized playing fields, and both outdoor and indoor facilities were used depending on the weather. The control group participated only in the traditional physical education activities planned for that semester, aimed at training various team and individual sports.

### Physical Fitness Testing

#### Countermovement Jump

Countermovement jump (CMJ) (21) height was measured using the Optojump system (Optojump photocell system; Microgate, Italy). The girls were instructed to swing their arms during the CMJ and to extend through their knees and ankles during the jump phase. After two familiarization jumps, participants had three attempts, with the best attempt included in the analysis. The intraclass correlation coefficient and coefficient of variation were 0.922 and 2.98 %, respectively.

#### Standing Long Jump

Participants were instructed to jump as far as possible and land with their feet together and in an upright position (22). The distance was measured in centimeters from the starting line to the heel of the participant. Initially, two familiarization jumps were performed, then participants had three attempts, with the best jump selected for analysis.

#### Overhead Medicine Ball Throw

Participants were instructed to hold a 2-kg medicine ball behind their head and throw it as far as possible above their head (23). The result was recorded in centimeters from the line of throw to the point of contact of the ball. They had three trials, with the best result being used in the analysis. The intraclass correlation coefficient and coefficient of variation were 0.841 and 3.91%, respectively.

#### Sit-Ups 30 S

The task was to perform as many complete sit-ups as possible in a 30 s time frame (24). The participant had to lie on his back with his knees bent and hands clasped behind his head. From this position, he had to rise as quickly as possible to a seated position and back to the starting position. The number of correctly performed sit-ups in 30 s was evaluated.

#### Bent Arm Hang

This test was used to assess muscular endurance of the upper limbs. Participants had to undergrip the bar and remain in a pull-up position with their chin above the bar for as long as possible (25). They had one attempt, which was measured in seconds.

#### Yo-Yo Intermittent Recovery Level 1 Test (YYIRT1)

YYIRT1 was performed according to the guidelines of Krustrup et al. (26). Participants had to run 2 × 20 m with 180° turns back and forth. After 40 m, participants jogged 2 × 5 m for active recovery. The audio device controlled the speed by beeps. The test was completed when a shuttle run was not finished within the sound signal. The total distance in meters is included in the analysis.

### Statistical Analysis

Data were analyzed using SPSS, version 23 (SPSS Inc., Chicago, IL, USA). The mean ± standard deviation was calculated for all results. Normality of data was assessed using Kolmogorov–Smirnov test and showed that all data were normally distributed (*p* > 0.05). Levene tests were also performed for all test variables. A two-way repeated measures ANOVA was calculated to test for main effects and interactions for time (baseline vs. post-intervention) and group (EXP vs. CON) on the selected outcomes. Effect size (ES) was tested using Cohen's d within each group according to Hopkins et al. (27) and was classified as < 0.2 (trivial); 0.2–0.6 (small); 0.6–1.2 (moderate); 1.2–2.0 (large); >2.0 (very large); and >4.0 (extremely large). In addition, partial Eta (η) squared (28) was applied to test the difference between the EXP and CON group [0.01 (small effect), 0.06 (moderate effect), and 0.14 (large effect)]. Significance was assumed at *p* ≤ 0.05.

## Results

Table 2 shows results of EXP and CON group. Results indicate a significant group (EXP vs. CON) × time (pre test vs. post test) interaction for standing long jump [*F*_(1, 57)_ = 41.994; *p* < 0.001], overhead medicine ball throw [*F*_(1, 57)_ = 18.224; *p* < 0.001], sit-ups 30 s [*F*_(1, 57)_ = 4.981; *p* = 0.030], bent arm hang [*F*_(1, 57)_ = 30.018; *p* < 0.001], and YYIRT1 score [*F*_(1, 57)_ = 9.230; *p* = 0.004]. Additionally, a significant main effect for time was found in countermovement jump [*F*_(1, 57)_ = 21.306; *p* < 0.001].

**Table 2:** Physical fitness results and and changes from pre- to post-test in EXP and CON group.

**Variable**	**Group**	**Pre-test**	**Post-test**	**ES**	**% Change**	***p*-value, **η**^2^*_***p***_***
Countermovement jump (cm)	EXP CON	31.9 ± 3.6 31.3 ± 4.4	34.3 ± 3.3 33.0 ± 5.3	+0.69 +0.35	+7.5 +5.4	Group: *p* = 0.343, η^2^*_*p*_*: 0.016 Time: *p* < 0.001, η^2^*_*p*_*: 0.272 Interaction: *p* = 0.489, η^2^*_*p*_*: 0.008
Standing long jump (cm)	EXP CON	171.1 ± 13.8 176.8 ± 12.0	178.2 ± 12.6 174.3 ± 11.9	+0.54 −0.21	+4.1 −1.4	Group: *p* = 0.787, η^2^*_*p*_*: 0.001 Time: *p* = 0.003, η^2^*_*p*_*: 0.146 Interaction: *p* < 0.001, η^2^*_*p*_*: 0.424
Overhead medicine ball throw (m)	EXP CON	6.2 ± 1.0 5.9 ± 1.1	6.5 ± 1.1 5.8 ± 1.2	+0.29 −0.09	+4.8 −1.7	Group: *p* = 0.084, η^2^*_*p*_*: 0.051 Time: *p* = 0.022, η^2^*_*p*_*: 0.089 Interaction: *p* < 0.001, η^2^*_*p*_*: 0.242
Sit-ups 30s (score)	EXP CON	18.1 ± 5.3 19.7 ± 4.2	19.2 ± 3.7 19.3 ± 4.2	+0.24 −0.10	+6.1 −2.0	Group: *p* = 0.435, η^2^*_*p*_*: 0.011 Time: *p* = 0.208, η^2^*_*p*_*: 0.028 Interaction: *p* = 0.030, η^2^*_*p*_*: 0.080
Bent arm hang (seconds)	EXP CON	39.9 ± 14.5 32.7 ± 10.2	42.4 ± 14.6 32.1 ± 10.2	+0.17 −0.06	+6.1 −1.8	Group: *p* = 0.009, η^2^*_*p*_*: 0.113 Time: *p* = 0.002, η^2^*_*p*_*: 0.158 Interaction: *p* < 0.001, η^2^*_*p*_*: 0.345
YYIRT1 (meters)	EXP CON	964.7 ± 242.3 947.6 ± 286.2	1066.7 ± 265.9 994.5 ± 290.9	+0.40 +0.16	+10.6 +4.9	Group: *p* = 0.527, η^2^*_*p*_*: 0.007 Time: *p* < 0.001, η^2^*_*p*_*: 0.542 Interaction: *p* = 0.004, η^2^*_*p*_*: 0.139

*EXP, experimental group; CON, control group; ES, Cohen d effect size; YYIRT1, Yo-Yo intermittent recovery test level 1. Values are defined as mean ± standard deviation*.

## Discussion

Early adolescence could be considered crucial for the development of physical activity behavior, especially in girls, as there is a significant decline in moderate- to-vigorous intensity of physical activity between the ages of 11 and 14 (29, 30). Hence, there is a call for interventions that could promote physical activity in adolescent girls. Recent discussion has suggested playing various team sports that have a positive impact on adolescent health (31). Therefore, the purpose of the current study was to determine the effects of a 12-week game-based school intervention on adolescent girls' physical fitness. The main findings of the current study were the improvement in aerobic fitness in the experimental group after a relatively short program. In addition, our game-based program over 12 weeks improved musculoskeletal fitness with the exception of CMJ, where there were no differences between groups.

Low aerobic fitness is associated with various cardiovascular diseases (32). Previous studies have shown positive effects on YYIRT1 performance in prepubertal and adolescent school children (20, 33–35). The current results suggest that a game-based school intervention is effective in improving aerobic fitness, as YYIR1 performance improved by 10.6%. A similar study using ball training showed no significant differences between groups for YYIRT1 performance after 10 months (33). However, due to the long intervention and already high levels of YYIRT1 performance in some participants, an additional median-split analysis showed positive effects in the participants with the poorest baseline levels. Two other studies showed smaller improvements than in our study after school-based volleyball program (2.4%) (35) and recreational soccer program (2.2%) (34). However, baseline YYIRT1 scores (947–964 m) were significantly lower in the current study compared to the aforementioned studies (1,292–1,504 m). Therefore, a possible discrepancy in the results could be due to the differences in baseline scores as well as the poor baseline scores of the participants in the current study. As for the differences between the EXP group and the CON group in the current study, it could be hypothesized that greater improvements occurred in the EXP group because team sports games were found to be more enjoyable compared to physical education classes, which could have an impact on participants' motivation during the program (35, 36).

The results for the vertical jump are contradictory. The CMJ was improved by +7.5% in the current study, but without significant differences between groups. Similar results were obtained after 8 months of recreational training in small-sided volleyball, in which the CMJ improved by 3.0%. Furthermore, Hammami et al. (36) found small effects on jumping performance in untrained adolescents after 8 weeks of soccer training. This was confirmed by Trajković et al. (34) following a school-based recreational soccer intervention, which showed only small effects (3.5%). In contrast, recreational soccer training in overweight and obese children showed significantly greater effects on CMJ (17.0%) (37). Differences in the duration of the interventions across studies, as well as differences in weight status and other characteristics, could be a possible explanation for this discrepancy between studies. There is also the possibility that different protocols were used to test vertical jump height, which could also contribute to differences in results.

Similar and conflicting results were found for the standing long jump. Sozen (38) found no significant differences in standing long jump after the volleyball school program compared to the control group. However, the regular physical education classes combined with the additional volleyball program promoted positive effects in the standing long jump (39). Similarly, Michailidis et al. (40) found significant improvement in standing long jump after 1 year of soccer training in 8–10 and 10–12 year old school children. In the present study, improvement in standing long jump (+4.1%) was found after 12 weeks of additional game-based activities. Significant changes between the initial and final measurements, as well as between groups, also occurred in explosive strength (medicine ball throw), upper body strength (bent arm hang), and strength endurance (sit-ups). It could be speculated that the significant differences between groups were due to the high number of high intensity activities during the team sports games. In addition, elements such as swings in volleyball and throws in handball and basketball could contribute significantly to the improvement of arm and shoulder muscle strength, thus improving explosive strength during throws. In addition, playing games requires a lot of activities involving changes of direction with the help of the trunk muscles (40), which could be the reason for the better improvement of the abdominal muscles compared to the control group.

Although we found significant improvements in physical fitness, the study has some limitations. We used only adolescent girls in the current study. Further studies should include both genders to show whether boys and girls receive the same benefits from this type of additional training in the school setting. In addition, we did not monitor students' overall physical activity during the intervention period or food intake, which could affect the overall results. Given that this is the age at which a significant decline in moderate-to-vigorous physical activity occurs, the greatest strength of the study is that a game-based after-school intervention provides an adequate incentive to improve physical fitness in adolescent girls compared to regular physical education.

## Conclusion

The results of this study show that the after-school game-based intervention significantly improves the physical fitness of adolescent girls compared to regular physical education. The most important finding is the improvement in aerobic fitness following game-based activities after a relatively short program. Significant improvement was also found in musculoskeletal fitness. The overall conclusion suggests that as few as two additional sessions per week are sufficient to produce significant changes in physical fitness in adolescent girls. Therefore, these results support the proposal to include additional forms of physical activity in school curricula.

## Data Availability Statement

The raw data supporting the conclusions of this article will be made available by the authors, without undue reservation.

## Ethics Statement

The studies involving human participants were reviewed and approved by Ethical Committee of Faculty of Sport and Physical Education Novi Sad (Ref. No. 11/2019). Written informed consent to participate in this study was provided by the participants' legal guardian/next of kin.

## Author Contributions

NT designed the research and recruited participants. ŠB conducted the data analysis and interpreted the results. TP drafted the manuscript. All authors read and approved the final version of the manuscript.

## Funding

This research was funded by Slovenian Research Agency (postdoctoral research project Z3-3212) and ARRS Research and Infrastructure Programme number P3-0323 (Renal Diseases and Renal Replacement Therapy).

## Conflict of Interest

The authors declare that the research was conducted in the absence of any commercial or financial relationships that could be construed as a potential conflict of interest.

## Publisher's Note

All claims expressed in this article are solely those of the authors and do not necessarily represent those of their affiliated organizations, or those of the publisher, the editors and the reviewers. Any product that may be evaluated in this article, or claim that may be made by its manufacturer, is not guaranteed or endorsed by the publisher.
